# Acute ketamine dysregulates task-related gamma-band oscillations in thalamo-cortical circuits in schizophrenia

**DOI:** 10.1093/brain/awy175

**Published:** 2018-07-17

**Authors:** Tineke Grent-‘t-Jong, Davide Rivolta, Joachim Gross, Ruchika Gajwani, Stephen M Lawrie, Matthias Schwannauer, Tonio Heidegger, Michael Wibral, Wolf Singer, Andreas Sauer, Bertram Scheller, Peter J Uhlhaas

**Affiliations:** 1 Institute of Neuroscience and Psychology, University of Glasgow, Glasgow, UK; 2 Department of Education, Psychology and Communication, University of Bari Aldo Moro, Bari, Italy; 3 Institute of Biomagnetism and Biosignalanalysis, University of Muenster, Germany; 4 Institute of Health and Wellbeing, University of Glasgow, UK; 5 Department of Psychiatry, University of Edinburgh, UK; 6 Department of Clinical Psychology, University Edinburgh, UK; 7 Department of Neurology, Goethe University, Frankfurt am Main, Germany; 8 MEG-Unit, Goethe University, Frankfurt am Main, Germany; 9 Department of Neurophysiology, Max Planck Institute for Brain Research, Frankfurt am Main, Germany; 10 Ernst Strüngmann Institute for Neuroscience (ESI) in Cooperation with Max Planck Society, Frankfurt am Main, Germany; 11 Frankfurt Institute for Advanced Studies (FIAS), Frankfurt am Main, Germany; 12 Department of Anaesthesia, Intensive Care Medicine and Pain Therapy, Goethe University, Frankfurt am Main, Germany

**Keywords:** schizophrenia, magnetoencephalography, *N*-methyl-d-aspartate receptor, neural oscillations, functional connectivity

## Abstract

Hypofunction of the *N*-methyl-d-aspartate receptor (NMDAR) has been implicated as a possible mechanism underlying cognitive deficits and aberrant neuronal dynamics in schizophrenia. To test this hypothesis, we first administered a sub-anaesthetic dose of S-ketamine (0.006 mg/kg/min) or saline in a single-blind crossover design in 14 participants while magnetoencephalographic data were recorded during a visual task. In addition, magnetoencephalographic data were obtained in a sample of unmedicated first-episode psychosis patients (*n = *10) and in patients with chronic schizophrenia (*n = *16) to allow for comparisons of neuronal dynamics in clinical populations versus NMDAR hypofunctioning. Magnetoencephalographic data were analysed at source-level in the 1–90 Hz frequency range in occipital and thalamic regions of interest. In addition, directed functional connectivity analysis was performed using Granger causality and feedback and feedforward activity was investigated using a directed asymmetry index. Psychopathology was assessed with the Positive and Negative Syndrome Scale. Acute ketamine administration in healthy volunteers led to similar effects on cognition and psychopathology as observed in first-episode and chronic schizophrenia patients. However, the effects of ketamine on high-frequency oscillations and their connectivity profile were not consistent with these observations. Ketamine increased amplitude and frequency of gamma-power (63–80 Hz) in occipital regions and upregulated low frequency (5–28 Hz) activity. Moreover, ketamine disrupted feedforward and feedback signalling at high and low frequencies leading to hypo- and hyper-connectivity in thalamo-cortical networks. In contrast, first-episode and chronic schizophrenia patients showed a different pattern of magnetoencephalographic activity, characterized by decreased task-induced high-gamma band oscillations and predominantly increased feedforward/feedback-mediated Granger causality connectivity. Accordingly, the current data have implications for theories of cognitive dysfunctions and circuit impairments in the disorder, suggesting that acute NMDAR hypofunction does not recreate alterations in neural oscillations during visual processing observed in schizophrenia.

## Teaser

NMDA receptor deficits have been implicated in schizophrenia. Grent-’t-Jong *et al.* show that treating healthy controls with ketamine triggers changes in neural oscillations during visual processing distinct from those seen in patients with schizophrenia. This suggests that acute NMDA receptor hypofunction does not account for visuo-perceptual deficits in the disorder.

## Introduction

Dysfunctions in cognitive and perceptual processes are a pervasive feature of schizophrenia that strongly impact on functional outcomes in the majority of patients (Green, 1996). Because no effective treatments are currently available that improve cognitive deficits, identification of circuit mechanisms that are amenable to novel pharmacological and behavioural interventions is a key priority of current research.

One hypothesis that has received considerable attention is the possibility that cognitive impairments in schizophrenia are the results of an altered balance between excitation and inhibition (Lewis and Moghaddam, 2006; Uhlhaas and Singer, 2012; Krystal *et al.*, 2017). During normal brain functioning, effective inhibition is mediated by different classes of γ-aminobutyric acid (GABA)ergic interneurons (Tremblay *et al.*, 2016) that regulate the activity of pyramidal cells through controlling their spike-timing and synchronization (Cobb *et al.*, 1995; Whittington *et al.*, 1995). This coordinated interplay leads to the emergence of rhythmic fluctuations in excitability or neural oscillations (Wang and Buzsaki, 1996; Traub *et al.*, 2004; Sohal *et al.*, 2009) that establish coherent information transfer between and within brain regions that underlies cognition and behaviour (Fries, 2015).

Recent evidence has assigned a critical role to the *N*-methyl-d-aspartate receptor (NMDAR) in the explanation of cognitive deficits and impaired neural dynamics in schizophrenia (Kantrowitz and Javitt, 2010; Anticevic *et al.*, 2012*b*; Gonzalez-Burgos and Lewis, 2012). At the circuit level, blockade of NMDARs leads to a reduction of interneuron activity but an increase in firing rates of pyramidal cells (Homayoun and Moghaddam, 2007), suggesting a disinhibition of neural outputs that could underlie the cognitive deficits observed following acute NMDAR administration.

Several mechanisms for changes in excitation and inhibition balance have been proposed, which include a specific role of NMDARs on parvalbumin-positive cells (Kinney *et al.*, 2006), but also other subclasses of interneurons (Jadi *et al.*, 2016) as well as presynaptic effects of NMDARs (Pafundo *et al.*, 2018). The potential direct and indirect interactions of NMDARs with parvalbumin-positive cells is of particular interest as parvalbumin-positive interneurons are prominently involved in the generation of gamma-band (30–100 Hz) oscillations through rhythmic inhibition of pyramidal cells (Sohal *et al.*, 2009) and have been found to be reduced in schizophrenia post-mortem tissue (Hashimoto *et al.*, 2008).

At the macroscopic scale, invasive electrophysiological data indicate that acute administration of NMDAR antagonists, such as ketamine, lead robustly to elevated gamma-band power in cortical (Pinault, 2008) and subcortical structures (Hakami *et al.*, 2009; Kocsis, 2012). In contrast, low-frequency activity at delta and theta-bands show less consistent effects with evidence for both up- and downregulation of oscillatory activity following ketamine administration but also other NMDA-antagonists, such as MK-801 (Hunt and Kasicki, 2013). However, there are data to suggest that the upregulation of high-frequency oscillations is observed predominantly during acute NMDAR hypofunction, while chronic administration of ketamine, for example, leads to a reduction of gamma-band oscillations (McNally *et al.*, 2013; Ahnaou *et al.*, 2017).

The modulation of neural oscillations through NMDAR antagonists and its compatibility with findings in schizophrenia is important as aberrant rhythmic activity, especially at gamma-band frequencies, has been implicated as a potential pathophysiological mechanism in the disorder that could account for the pervasive cognitive deficits and certain symptoms (Uhlhaas and Singer, 2010; Lewis *et al.*, 2012). Specifically, there is evidence to suggest that acute administration of ketamine reproduces many of the clinical symptoms of schizophrenia and cognitive deficits in healthy volunteers (Krystal *et al.*, 1994) as well as impairments in electrophysiological parameters, such as in mismatch negativity (Umbricht *et al.*, 2000; Schmidt *et al.*, 2013) and corollary discharge (Kort *et al.*, 2017). More recent evidence has also highlighted that physiological alterations induced by ketamine in functional MRI (Anticevic *et al.*, 2012*b*; Kraguljac *et al.*, 2017) may resemble the alterations in resting state data observed in early stage schizophrenia but not in chronic schizophrenia (Anticevic *et al.*, 2015*a*), suggesting that acute NMDAR hypofunctioning could provide a pharmacological model relevant for understanding the pathophysiology of schizophrenia with potentially important treatment implications. Accordingly, it is critical to examine whether the spectral signatures induced by NMDAR antagonists, such as ketamine, are compatible with electrophysiological alterations observed in schizophrenia to further validate the NMDAR hypothesis of schizophrenia.

To systematically test this hypothesis, we investigated the effects of a sub-anaesthetic dose of ketamine on gamma band (30–100 Hz) oscillations and functional connectivity in a visual paradigm that elicits robust high-frequency oscillations with excellent test-retest reliability in controls (Hoogenboom *et al.*, 2006; Tan *et al.*, 2016). In addition, we also assessed activity at theta (4–8 Hz), alpha (8–12 Hz) and beta (13–30 Hz) bands and directed functional connectivity in thalamo-cortical circuits. Previous preclinical data (Dawson *et al.*, 2013; Anderson *et al.*, 2017) and studies with human participants (Rivolta *et al.*, 2015) have indicated that NMDARs prominently dysregulate activity in thalamo-cortical networks. Moreover, in schizophrenia, both functional (Woodward *et al.*, 2012; Anticevic *et al.*, 2015*b*) as well as anatomical alterations in thalamo-cortical circuits (Wagner *et al.*, 2015) have been observed, highlighting the importance of further identifying pathophysiological mechanisms that give rise to aberrant information processing.

Ketamine-induced changes in spectral power and functional connectivity were compared with magnetoencephalography (MEG) data obtained from a sample of patients with chronic schizophrenia as well as patients experiencing their first-episode of psychosis (FEP) to allow for a comparison with altered neuronal dynamics induced by NMDAR hypofunctioning. Based on extensive evidence highlighting reductions of sensory-induced gamma band oscillations during auditory and perceptual processing in schizophrenia (Kwon *et al.*, 1999; Spencer *et al.*, 2008; Sun *et al.*, 2013; Grent-’t-Jong *et al.*, 2016; Thune *et al.*, 2016), we hypothesized that acute administration of ketamine in healthy volunteers would lead to a reduction in the power of task-related gamma-band oscillations. In addition, we expected that ketamine would recreate patterns of functional connectivity in schizophrenia and FEP, providing further support for the NMDAR hypothesis of schizophrenia.

## Materials and methods

### Participants

Three samples of participants were recruited for this study. The first group comprised 14 healthy participants who were administered a sub-anaesthetic dose of ketamine. The study followed a single-blind, randomized, placebo-controlled, crossover design. An experimental session started with either a bolus injection of 10 mg S-ketamine (ketamine condition) or 10 ml of NaCl 0.9% (placebo condition). This was followed by continuous intravenous infusion of 0.006 mg S-ketamine per kg body weight per minute in the ketamine or continuous infusion of NaCl 0.9% in the placebo condition. During the 45 min of continuous infusion, participants performed a visual task that robustly elicits high-frequency oscillations (Hoogenboom *et al.*, 2006). MEG data obtained during resting state recordings have been published previously (Rivolta *et al.*, 2015). Following the MEG recordings, the Positive and Negative Syndrome Scale (PANSS) (Kay *et al.*, 1987) was administered. Patients were also rated on the item ‘inappropriate affect’, which allowed for a score on the factor ‘disorganization’ (Cuesta and Peralta, 1995). Screening procedures involved ECG, vital signs, blood test, drug tests and the Structured Clinical Interview for DSM (SCID) for possible signs of psychosis.

The second group comprised a sample of antipsychotic, medication-naïve patients with FEP (*n = *10: *n = *8 schizophrenia, *n = *1 schizoaffective disorder and *n* = 1 schizophreniform disorder), recruited from NHS services in Glasgow and Edinburgh as well as from the general population. Ten age- and gender-matched healthy control subjects were recruited from the local community (Table 1).

**Table 1:** Demographic, behavioural and psychopathological data

	Placebo	SEM	Ketamine	SEM	Statistics
Age, years	29 ± 0.9				–
Gender	12 male / 2 females				–
**PANSS scores**					
Negative	8.0	0.6	13.5	1.0	*t*(13) = 5.0, *P < *0.0001
Excitement	5.4	0.3	6.8	0.5	*t*(13) = 2.9, *P < *0.0015
Cognitive	5.4	0.3	10.5	0.7	*t*(13) = 7.4, *P < *0.0001
Positive	4.1	0.1	7.2	0.5	*t*(13) = 6.8, *P < *0.0001
Depression	5.6	0.2	10.5	0.4	*t*(13) = 10.7, *P < *0.0001
Disorganization	3.1	0.1	5.8	0.6	*t*(13) = 4.9, *P < *0.0001
Total	35.7	0.9	60.1	2.3	*t*(13) = 11.6, *P < *0.0001
**Behaviour**					
Mean RT, ms	564	16.8	645	18.1	*F*(1,13) = 18.4, *P = *0.001
Accuracy, % hits	92.5	1.1	68.7	3.3	*F*(1,13) = 71.6, *P < *0.0001
**MEG trials, *n***	349	20.6	353	19.2	n.s.
			
	**Controls**		**FEP**	
Age, years	23.1	1.0	23.2	1.3	n.s.
Gender	5 males / 5 females		5 males / 5 females		–
**PANSS scores**					^a^
Negative	–	–	13.8	0.9	n.s.
Excitement	–	–	7.7	1.1	n.s.
Cognitive	–	–	18.0	2.0	*t*(23) = 3.9, *P = *0.002
Positive	–	–	19.1	2.1	*t*(23) = 3.9, *P = *0.002
Depression	–	–	12.0	1.5	n.s.
Total	–	–	70.6	5.5	*t*(23) = 2.9, *P = *0.011
**Behaviour**					
Mean RT, ms	514	20.6	575	28.6	*F*(1,9) = 5.5, *P = *0.044
Accuracy, % hits	92.9	1.2	87.1	3.6	n.s.
**MEG trials, *n***	225	6.3	221	9.4	n.s.
			
	**Controls**		**Chronic schizophrenia**	
Age, years	30.8	8.0	34.1	10.6	n.s.
Gender	10 males / 6 females		10 males / 6 females		–
**PANSS scores**					–
Negative	–	–	14.3	1.6	–
Excitement	–	–	6.9	0.7	–
Cognitive	–	–	9.6	0.7	–
Positive	–	–	9.9	1.0	–
Depression	–	–	11.3	0.7	–
Total	–	–	51.9	3.4	–
**Behaviour**					
Mean RT, ms	572	52.0	596	50.0	n.s.
Accuracy, % hits	94.7	1.0	82.5	3.1	*F*(1,15) = 129.3, *P < *0.001
**MEG trials, *n***	322	23.6	336	27.9	n.s.

^a^Independent-sample *t*-tests, 1000 samples bootstrapping applied: PANSS scores, FEP versus schizophrenia.

n.s. = not significantly different; RT = response times.

The third group comprised 16 patients with chronic schizophrenia recruited from the in- and outpatients units of the Department of Psychiatry, Goethe-University, Frankfurt am Main, Frankfurt, Germany. All schizophrenia patients met DSM-IV criteria for schizophrenia and were on stable antipsychotic treatment. Analysis of spectral power data from this sample were published previously (Grent-‘t-Jong *et al.*, 2016). Sixteen age- and gender-matched healthy controls were recruited from the local community (Table 1).

The study was carried out according to the Declaration of Helsinki and approved by the ethical committees of the Goethe University Frankfurt, Germany, and NHS Greater Glasgow and Clyde. Written informed consent was obtained after participants received and agreed to a complete description of the study protocol. The study was registered as a clinical trial (PHARMASYN; EudraCT-Nr: 2011-002937-21).

### Stimuli and task

**Figure 1:**
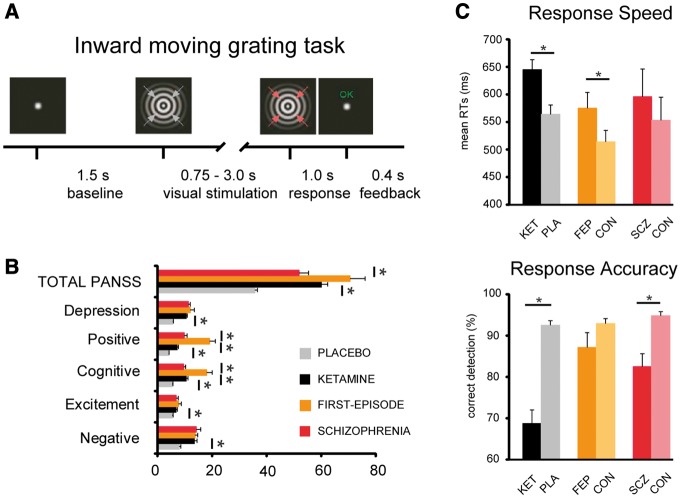
**Psychopathology and behavioural performance. **(**A**) Paradigm: participants detect increase in speed of inward moving grating (indicated by red arrows), after which the trial ends and feedback is provided. (**B**) Means and standard error of means for scores on PANSS ratings. Ketamine significantly increased all PANSS ratings (*P* < 0.0015 for excitement subitems and *P* < 0.001 for all others). Largest increases in symptom severity were seen for cognitive and positive symptoms (also expressed in total scores) in ketamine versus placebo condition and first-episode versus schizophrenia patients and FEP patients versus ketamine condition (all *P* < 0.001). (**C**) Mean response times (RTs, *top*) and response accuracy (*bottom*) during the inward moving grating task for the different conditions/groups. Significantly slower responses were recorded for ketamine compared to placebo in healthy individuals (*P* = 0.001), and between first-episode patients and their controls (*P* = 0.044). Response accuracy was significantly lower after ketamine infusion (*P* < 0.0001) compared to placebo, and in patients with chronic schizophrenia compared to controls (*P* < 0.001).

The visual task paradigm (Fig. 1A) was modified after Hoogenboom *et al.* (2006). On each trial, participants received an inward moving circular sine wave grating (visual angle: 5°; spatial frequency: two cycles per degree; contrast: 100%). An LCD projector located outside the magnetically shielded MEG room projected the stimuli onto the screen via two front-silvered mirrors, controlled by Presentation software (Neurobehavioral Systems). The participants’ task was to press a response button after detecting a speed increase of the inward moving grating, which occurred randomly between 750 and 3000 ms post-stimulus onset (no-acceleration catch trials occurred on ∼5% of the trials). Response feedback was provided on the screen at the end of each trial.

### Neuroimaging

MEG data during ketamine administration and from patients with chronic schizophrenia were acquired using a 275-sensor whole-head system (Omega 2005, VSM MedTech Ltd) with a sampling rate of 600 Hz in a synthetic third order axial gradiometer configuration. For the FEP sample, MEG data were acquired, using a 248-channel 4D-BTI whole-head magnetometer system (MAGNES^®^ 3600 WH, 4D-Neuroimaging), recording at a sampling frequency of 1017.25 Hz, filtered online between DC and 400 Hz.

Recordings with movements >5 mm were excluded from the analysis. A high-resolution anatomical MRI scan was acquired for each participant on a 3 T Siemens Trio scanner, using a 3D-MPRAGE sequence (160 slices, voxel size: 1 × 1 × 1 mm; field of view: 256 mm, repetition time: 2300 ms, echo time: 3.93 ms), with markers placed at the same locations as the sensors used for recording head position in the MEG. These markers were used for subsequent co-registration of the MEG data to the anatomical T_1_ image.

### MEG data analysis

MEG data were analysed with MATLAB using the open-source Fieldtrip Toolbox (Oostenveld *et al.*, 2011). Trials were defined as non-overlapping data segments starting 1000 ms before stimulus onset until 1800 ms post-stimulus onset. Power line fluctuations were removed by using a discrete 50 Hz Fourier transform filter (including the first two harmonics). Trials containing muscle artefacts or sensor (SQUID) jumps were discarded using semi-automatic artefact rejection routines. Artefacts due to eye blinks, eye movements and the heartbeat signal were removed using independent component analysis decomposition and removal strategies. Furthermore, data were downsampled to 300 Hz.

All analyses were conducted on ‘virtual channel’ reconstructed MEG data. Linearly constrained minimum variance (LCMV) beamformer spatial filters (Van Veen *et al.*, 1997) were used to first reconstruct the MEG data from MNI source locations corresponding to centroids of 18 of 116 available AAL atlas regions (Tzourio-Mazoyer *et al.*, 2002). Fourteen regions were included in the power analyses: 12 covering striate and extra-striate visual-cortical areas based on prior findings (Hoogenboom *et al.*, 2006; Tan *et al.*, 2016) and two (bilateral) thalamic regions. For analyses of Granger causality connectivity, four additional nodes were included, covering left and right precuneus and posterior cingulate gyrus, posterior regions involved in attention and top-down control (Dosenbach *et al.*, 2008; Heilbronner and Platt, 2013).

Time-frequency power representations (TFRs) were computed on the 12 occipital and two thalamic LCMV reconstructed time-series, using a sliding window Fast Fourier Transform (FFT) approach with a fixed window of 225 ms and a step size of 50 ms across the length of the epochs. Power of all frequencies between 1 and 90 Hz were estimated based on data padded up to the 4 s, using a frequency resolution of 1 Hz, and multiplying the data with a Hanning taper before averaged power estimation. All analyses focused on task activity from the latency window between the initial evoked response and first potential speed change of the stimulus (350–750 ms). Statistically significant frequency ranges for condition and group effects (see below) were estimated unbiased statistically from the full range of estimated frequencies (1–90 Hz).

Analyses first focused on contrasting task-induced activity (350–750 ms post-stimulus) with prestimulus activity (−500 to −100 ms) separately for ketamine and placebo conditions (Task effects). Subsequently, Condition effects of ketamine were tested directly by contrasting with TFR data from the placebo condition. For the FEP and schizophrenia groups, baseline-corrected task-induced activity was contrasted with data from their matched control participants (Group effects).

To address changes in directed functional connectivity, Granger causality estimates were computed across all 18 nodes. Granger causality is a technique used to assess directed, functional interactions between different brain regions and provides directional information about interareal communication (Seth *et al.*, 2015). Granger causality estimates were computed using a non-parametric approach, including spectral density matrices estimated directly from Fourier transformed data (350–750 ms) in the 0–149 Hz range (Hanning tapered, frequency smoothing of 4 Hz, padded to 4 s), followed by matrix factorization and variance decomposition. For complex spectral data, this approach has the advantage of fewer assumptions and lacks the shortcomings of parametric approaches using autoregressive modelling (Mitra and Pesaran, 1999; Dhamala *et al.*, 2008).

To determine the alterations in feedforward (FF) versus feedback (FB) activity, we computed the directed asymmetry index (DAI; Bastos *et al.*, 2015; Michalareas *et al.*, 2016) for all significant projections, using the following formula:

(1) DAI(AtoB)=[GC(AtoB)−GC(BtoA)]/[GC(AtoB)+GC(BtoA)]

A positive value indicates that the connection A to B is predominantly feedforward, whereas a negative DAI indicates that the flow of information is predominantly feedback. The human Granger causality (GC)-derived DAI index obtained from MEG data has been shown to correlate strongly with SLN values derived from macaque retrograde tracing data (SLN equals the number of supragranular neurons normalized by the number of supra- and infragranular neurons that give rise to a projection from areas AtoB) (Michalareas *et al.*, 2016).

### Statistical analysis

Both condition-specific Task effects of ketamine and placebo and their difference (Condition effects: ketamine versus placebo), were statistically evaluated for data across the 12 occipital cortex regions of interest, and separately, for the left and right thalamus, using data averaged over time points of interest. Statistical testing included non-parametric Monte-Carlo permutation dependent *t*-test statistics (1500 permutations) and cluster-based correction for multiple comparisons (*P <* 0.05, two sided).

Granger causality estimates were tested using a similar statistical approach, including Granger causality data averaged within two frequency bins: (i) a low-frequency (5–28 Hz) range; and (ii) gamma-band range (63–80 Hz), corresponding to the frequencies of significant effects of ketamine on power estimates. For the group comparisons between FEP and schizophrenia groups versus controls, independent *t*-tests were used. Output of the first-level permutation statistics were in addition false discovery rate (FDR)-controlled for type I errors by accepting only those connections that exceeded the number of significant connections (*n* = 15) expected by chance at our alpha level of 0.05. Furthermore, changes in PANSS score estimates were assessed using paired-sample *t*-tests for ketamine versus placebo data and independent-sample *t*-tests (both 1000 samples bootstrapped) for the contrast between FEP and schizophrenia groups. Behavioural performance was tested with repeated-measures ANOVA in a within-subject design.

Finally, to assess Group effects, data from each main group (ketamine, FEP, schizophrenia) were *z*-normalized with respect to data from their respective control group and submitted to a one-way ANOVA using 1000 samples bootstrapping, 95% confidence interval (CI), and least significant difference (LSD) for multiple correction of *post hoc* pairwise comparisons.

#### Effect sizes

Cohen’s *d* effect sizes were computed for both power and Granger causality analyses, separately per group. For the ketamine group, these were computed as the difference between the means of the ketamine and placebo conditions, divided by the pooled standard deviation (SD) across conditions. For the patient groups, effect sizes were obtained through the difference between the means of the patient group and their respective controls, divided by the control group’s SD.

### Data availability

The datasets generated during and/or analysed during the current study are available from the corresponding author on reasonable request.

## Results

### Behavioural data: ketamine

PANSS data (*n = *14) showed that ketamine significantly increased scores on all six subscales (Fig. 1B and Table 1). Behavioural responses (Fig. 1C) indicated that ketamine administration significantly increased response times [*F*(1,13) = 18.4, *P = *0.001, mean response times placebo = 564 ± 16.8 ms (SEM) and ketamine = 645 ± 18.1 ms] while significantly decreasing accuracy [*F*(1,13) = 71.6, *P < *0.0001, placebo = 92.5 ± 1.1%, ketamine = 68.7 ± 3.3%].

### Behavioural and psychopathology data: FEP and chronic schizophrenia

The FEP group had significantly elevated total PANSS ratings and increased scores on the ‘Positive’ and ‘Cognitive’ factors compared to chronic schizophrenia patients (Fig. 1B and Table 1). Furthermore, additional one-way ANOVAs revealed that the elevated PANSS Positive and Cognitive factor scores in patients with FEP, but not in patients with schizophrenia, differed significantly (*P < *0.05, Bonferroni corrected) from those observed following ketamine administration in healthy individuals [Group × PANSS Cognitive: *F*(2,38) = 15.3, *P < *0.0001, Group × PANSS Positive: *F*(2,38) = 24.6, *P < *0.0001].

Behavioural performance (Fig. 1C) in patients with FEP and with chronic schizophrenia was impaired compared to their age- and gender-matched healthy control participants (Table 1). The FEP group had slower response times [*F*(1,9) = 5.5, *P = *0.044, mean response times ± standard error of the mean (SEM) FEP = 575 ± 28.6 ms and Controls = 514 ± 20.6 ms], whereas patients with schizophrenia were characterized by reduced response accuracy [*F*(1,15) = 129.3, *P < *0.001, schizophrenia = 82.5 ± 3.1%, Controls = 94.7 ± 1.0%]. At the second-level, mean response times and response accuracy were similar across groups.

### MEG data

#### Ketamine spectral power

**Figure 2:**
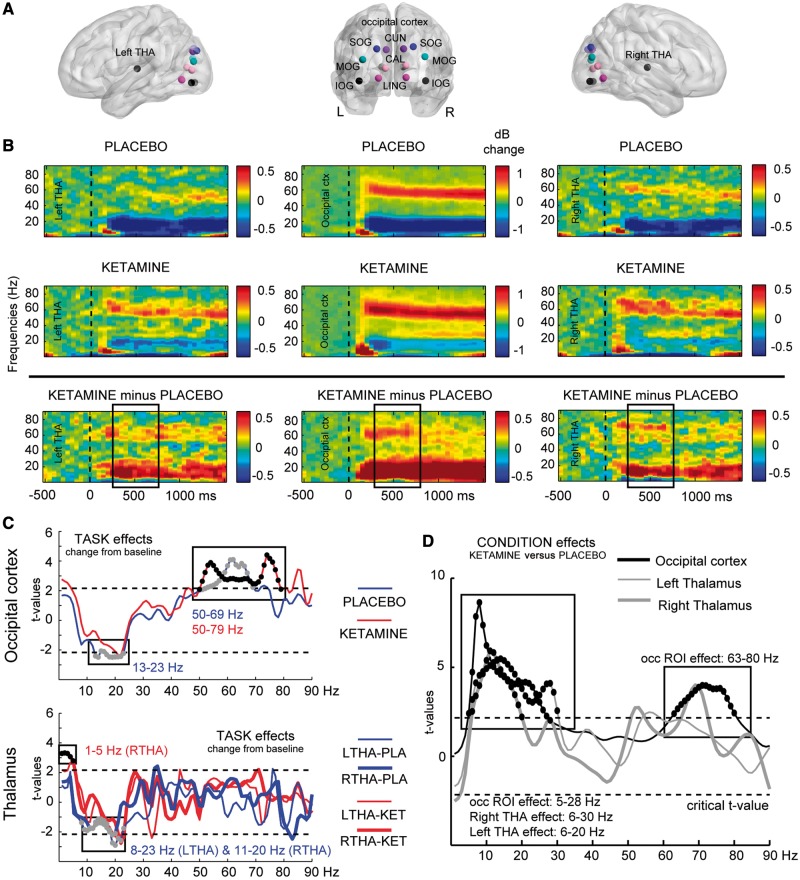
**Effects of ketamine on spectral power. **(**A**) Indication of regions of interest (ROIs) used in the analysis, projected onto a transparent MNI template brain. CAL = calcarine sulcus; CUN = cuneus; LING = lingual gyrus; SOG/MOG/IOG = superior/middle/inferior occipital gyrus; THA = thalamus. (**B**) Time–frequency responses (TFRs) of AAL atlas reconstructed virtual channel data. *Middle* panel shows grandaverage (*n = *14) data from across 12 regions in the occipital cortex (averaged dB power changes from baseline), whereas the *left* and *right* panels show the response in left and right thalamus, respectively. Data are time-locked to the onset of the visual grating stimulus. These TFRs indicate the effect of Task on brain oscillations, separately for the placebo (*first row*) and ketamine (*second row*) condition, as well as their difference (*third row*). Boxes placed over the *third row* TFRs indicate the window of interest in the analyses (350–750 ms). (**C**) Results of cluster-based permutation statistics of Task effects (change from baseline: 350 to 750 ms versus −500 to −100 ms) for both conditions separately (placebo in blue, ketamine in red) and separately for the occipital cluster (*top*) and left and right thalamus (*bottom*). Boxes (and black and grey dots) indicate *t*-values within significant frequency clusters (cluster corrected, *P < *0.05). (**D**) As in **C**, but for the Condition effects (ketamine versus placebo), using dB change from baseline data from each condition. In occipital regions, ketamine significantly upregulated both high- and low-frequency power (*d* = 0.88/1.12), whereas patients showed high-frequency power down regulation (FEP: *d* = 1.49, schizophrenia: *d* = 1.07), with upregulation of low-frequency power only for chronic patients (*d* = 1.06). In the thalamus, low frequency power was upregulated for both ketamine and chronic schizophrenia group (1.31 < *d* < 0.99), but not for the FEP group.

Condition-specific effects revealed significantly increased gamma-band power in the occipital regions of interest in both ketamine and placebo conditions, but with a slight difference in bandwidth extending into a higher gamma range under ketamine challenge (placebo: 50–69 Hz, *T_sum_* = 59.3, *P = *0.004; ketamine: 50–79 Hz, *T_sum_* = 91.3, *P = *0.002) (Fig. 2B, C and Table 2). A comparable spectral profile was observed in the left and right thalamus regions of interest (Fig. 2B). However, the spectral-power modulation in thalamic regions of interest was weaker than occipital regions and, as a result, the task effects were not significant at gamma-band frequencies (Fig. 2C).

**Table 2:** Regions of interest showing significant effects of ketamine (task and condition effects) and significant group differences (FEP/schizophrenia versus controls) for task-induced changes in power (1–90 Hz)

ROI	Contrast	Frequency range	T_sum_-value	*P-*value
**Task effects**				
Occipital ROI	Placebo: active versus baseline Ketamine: active versus baseline	13–23 Hz 50–69 Hz 50–79 Hz	−25.9 59.3 91.3	0.044 0.004 0.002
Left thalamus	Placebo: active versus baseline	8–23 Hz	−29.9	0.004
Right thalamus	Placebo: active versus baseline Ketamine: active versus baseline	11–20 Hz 1–5 Hz	−17.4 15.8	0.034 0.022
**Condition effects**				
Occipital ROI	Ketamine versus placebo	5–28 Hz 63–80 Hz	107.8 60.8	0.004 0.030
Left thalamus	Ketamine versus placebo	6–30 Hz	105.1	0.002
Right thalamus	Ketamine versus placebo	6–20 Hz	63.0	0.002
**Group effects**				
Occipital ROI	FEP versus controls Schizophrenia versus controls	53–76 Hz 62–83 Hz 7–23 Hz	−55.7 −57.9 46.9	0.046 0.026 0.032
Left thalamus	Schizophrenia versus controls	7–20 Hz	37.2	0.034
Right thalamus	Schizophrenia versus controls	8–20 Hz	34.7	0.028

Occipital region of interest (ROI) includes: bilateral calcarine fissure, cuneus, lingual gyrus, superior, middle and inferior occipital gyrus, T_sum_ = sum of all *t*-values of cluster of significant frequency bins, *P*-values are cluster corrected across regions of interest and frequencies.

In frequencies <50 Hz, the characteristic desynchronization at alpha/beta-band frequencies was found only for the placebo condition in occipital regions of interest (Fig. 2B and C; 13–23 Hz: *T_sum_* = − 25.9, *P = *0.044) and in the left and right thalamus [Fig. 2B and C; left thalamus (8–23 Hz), *T_sum_* = − 29.9, *P = *0.004, right thalamus (11–20 Hz), *T_sum_* = − 17.4, *P = *0.034]. In the ketamine condition, an additional increase in spectral power in the right thalamus in the 1–5-Hz range was observed (Fig. 2C; *T_sum_* = 15.8, *P = *0.022).

The contrast ketamine versus placebo condition confirmed the ketamine-induced shift to increased higher gamma band activity (63–80 Hz) in the occipital regions of interest (*T_sum_* = 60.8, *P = *0.03, *d* = 0.88), as well as the upregulation of low frequency activity in the alpha/beta range in both occipital channels (5–28 Hz: *T_sum_* = 107.8, *P = *0.004, *d* = 1.12), left thalamus (6–30 Hz: *T_sum_* = 105.1, *P = *0.002, *d* = 1.31) and right thalamus (6–20 Hz: *T_sum_* = 63.0, *P = *0.002, *d* = 0.99).

#### Ketamine functional connectivity

Ketamine induced widespread changes in Granger causality values between thalamo-cortical and cortico-cortical connections, with Granger causality patterns characterized by predominantly hypo-connectivity (Fig. 4 and Table 3), involving both lower (5–28 Hz) and higher frequencies (63–80 Hz).

**Table 3:** Summary of significant Granger causality effects for low (5–28 Hz) and high frequency (63–80 Hz) activity, separately for each experimental contrast (ketamine versus placebo, schizophrenia versus controls, and FEP versus controls

	Significant connections	*t*-values	*P*-values
**Ketamine versus placebo (*n = *14)**			
**Low frequency (5–28 Hz)**			
Thalamo-cortical connectivity	RPCUN-RTHA, LPCG-RTHA, LPCUN-LTHA, RTHA-RIOG	−2.2 to −3.4	0.0211 to 0.0001
Cortico-cortical connectivity	LLING-RIOG, LCUN-LCAL, LPCG-RIOG, LSOG-LLING, LSOG-LCAL, LIOG-RIOG, LMOG-RCUN, LMOG-RPCUN, LSOG-LCUN, LCAL-LCUN, LMOG-RLING, RIOG-LPCUN, LPCUN-RSOG, LMOG-RCAL, LIOG-LCAL, LMOG-RSOG, RLING-LCUN, RIOG-LSOG, LMOG-RPCG, RSOG-RCUN	−2.0 to −3.3	0.0271 to 0.0001
**High frequency (63–80 Hz)**			
Thalamo-cortical connectivity	RCAL-LTHA, RTHA-RPCUN	2.5 to 2.8	0.0198 to 0.0130
Cortico-cortical connectivity	LSOG-LPCUN, LLING-LPCUN, LIOG-LPCUN, RIOG-LMOG	−2.0 to −2.2	0.0213 to 0.0031
**FEP versus controls (*n = *10)**			
**High frequency (63–80 Hz)**			
Thalamo-cortical connectivity	LMOG-RTHA	2.8	0.0062
Cortico-cortical connectivity	RSOG-LPCG, RSOG-LIOG, RMOG-RPCUN	2.5 to 3.2	0.0077 to 0.0043
**Schizophrenia versus controls (*n = *16)**			
**Low frequency (5–28 Hz)**			
Thalamo-cortical connectivity	LPCUN-LTHA, RPCUN-LTHA	2.3 to 2.9	0.0190 to 0.0030
Cortico-cortical connectivity	RPCG-RIOG, RPCG-LPCUN, RPCG-LMOG, LPCG-RPCUN, LPCUN-RCUN, RPCUN-LMOG, RPCG-RCAL, RPCG-RMOG, LPCG-RSOG, LPCUN-RMOG, LPCG-LCAL, LPCUN-RLING, LPCUN-RCAL, LPCG-RIOG, LPCG-RLING, RPCG-RLING, LLING-LCAL, LPCG-RCAL, LPCG-RMOG	2.2 to 3.7	0.0170 to 0.0010
**High frequency (63–80 Hz)**			
Thalamo-cortical connectivity	RPCG-RTHA, LPCUN-LTHA, RPCUN-LTHA, RPCG-LTHA, RTHA-RLING	2.2 to 2.4	0.0200 to 0.0070
Cortico-cortical connectivity	RPCG-LMOG, LPCG-RSOG, RPCUN-RSOG, RPCG-LSOG, RPCG-LCUN, RPCUN-LMOG, RPCG-LLING, LPCG-RSOG, LPCG-RCUN, LPCG-RLING, RPCG-RLING, LPCG-RIOG	2.1 to 2.8	0.0250 to 0.0040

CAL = calcarine sulcus; CUN = cuneus; IOG = inferior occipital gyrus; LING = lingual gyrus; MOG = middle occipital gyrus; PCG = posterior cingulate cortex; PCUN = precuneus; SOG = superior occipital gyrus; THA = thalamus; L = left hemisphere; R = right hemisphere.

**Figure 3:**
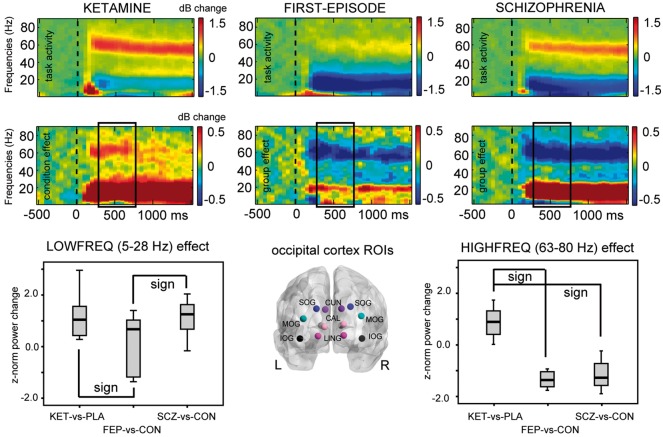
**Comparisons of spectral power changes between ketamine, FEP and chronic schizophrenia groups.** *Top* panels show time–frequency response plots indicating occipital regions of interest recorded task-effect of ketamine in healthy controls (*top left*) and the condition-effect between ketamine and placebo (*bottom left*), compared to task- and group-effects of similar task data recorded in FEP patients and controls (*middle*) and in patients with chronic schizophrenia and controls (*right column* plots). All data were averaged across the regions of interest indicated in the *middle bottom* panel, including data from left and right cuneus (CUN), calcarine fissure (CAL), lingual gyrus (LING), and superior/middle/inferior occipital gyrus (SOG/MOG/IOG). The *lower* panel also shows box-plots with data from each Group contrast, z-normalized to their respective control group [ketamine versus placebo (KETvsPLA), FEP versus controls (FEPvsCON), and schizophrenia versus controls (SCZvsCON)]. Main effects of Group contrast were found for both low frequency range (*P* = 0.035) and high frequency range effects (*P* < 0.0001), with significant *post hoc* pairwise comparisons indicated in the figures [low frequency: ketamine versus FEP (KETvsFEP), *P* = 0.016; FEP versus schizophrenia (FEPvsSCZ), *P* = 0.026; high-frequency: KETvsFEP and KETvsSCZ, *P* < 0.0001].

For thalamo-cortical interactions, ketamine significantly (*d* = 1.21) decreased connectivity in the lower-frequency range with the precuneus, posterior cingulate gyrus and inferior occipital gyrus. In the higher-frequency range, a significant increase (*d* = 0.71) was observed in thalamo-cortical connectivity with precuneus and calcarine fissure. Cortical-cortical connectivity was decreased in strength for both lower (*d* = 1.42) and higher frequencies (*d* = 0.64) across a wide range of occipital-parietal connections (Table 4).

**Table 4:** Summary table of main findings and Cohen’s *d* effect sizes

	Ketamine versus placebo	FEP versus controls	Schizophrenia versus controls
**Oscillatory power changes**			
Occipital cortex: 63–80/53–76/62–83 Hz	↑ 0.88	↓ 1.49	↓ 1.07
Occipital cortex: 5–28/7–23 Hz	↑ 1.12	−	↑ 1.06
Left thalamus: 6–20/7–20 Hz	↑ 1.31	−	↑ 1.07
Right thalamus: 6–30/8–20 Hz	↑ 0.99	−	↑ 1.02
**Granger causality connectivity changes**			
Low frequency (5–28 Hz)			
Thalamo-cortical connectivity	↓ 1.21	−	↑ 0.98
Cortico-cortical connectivity	↓ 1.42	−	↑ 1.24
High frequency (63–80 Hz)			
Thalamo-cortical connectivity	↑ 0.71	↑ 1.26	↑ 0.93
Cortico-cortical connectivity	↓ 0.64	↑ 1.40	↑ 1.01

In regards to the effect of ketamine on feedforward- versus feedback-mediated thalamo-cortical and cortico-cortical connectivity, we observed alterations for both feedforward and feedback processing at lower and high-frequencies (Supplementary Table 2).

#### Spectral power in chronic schizophrenia and FEP

Overall, the direction of both task-induced power changes in schizophrenia and FEP groups was different from those seen in healthy controls during ketamine infusion (Table 2 and Fig. 3). Gamma-band power in the occipital regions of interest was significantly decreased in both FEP and chronic schizophrenia groups [Table 2 (group effects): FEP versus controls: 53–76 Hz, *T_sum_* = − 55.7, *P = *0.046, *d* = 1.49; schizophrenia versus controls: 62–83 Hz, *T_sum_* = − 57.9, *P = *0.026, *d* = 1.07], whereas low-frequency power was significantly increased in patients with chronic schizophrenia, but not in patients with FEP, in occipital regions [Table 2 (Group effect): schizophrenia versus controls; 7–23 Hz, *T_sum_* = 46.9, *P = *0.032, *d* = 1.06] as well as in thalamic regions of interest (left thalamus: 7–20 Hz, *T_sum_* = 37.2, *P = *0.034, *d* = 1.07; right thalamus: 8–20 Hz, *T_sum_* = 34.7, *P = *0.028, *d* = 1.02).

**Figure 4:**
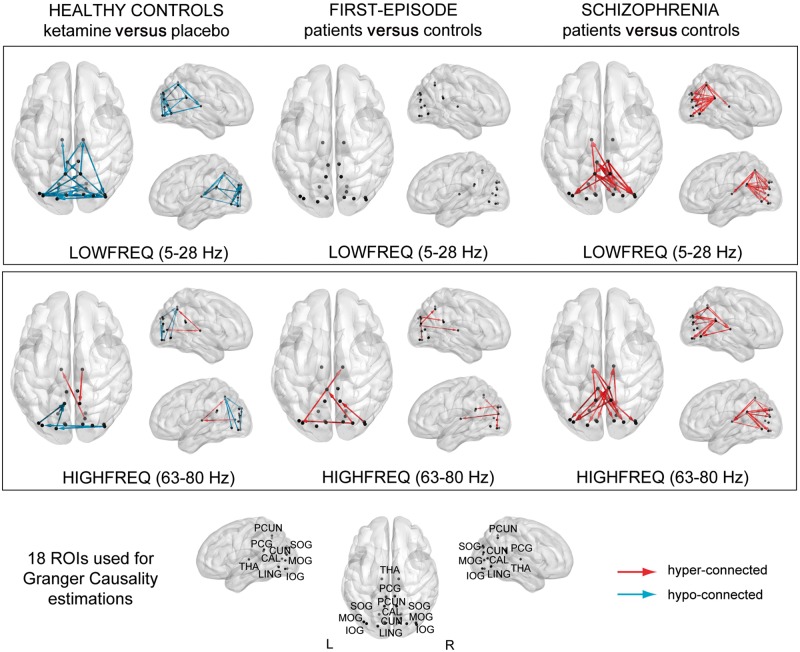
**Changes in functional connectivity in thalamo-cortical circuitry. **Statistically significant (*P < *0.05, Type I error corrected) Granger causality changes between ketamine and placebo condition in healthy controls (*left*), FEP patients and controls (*middle*) and chronic schizophrenia patients and controls (*right*) during stimulus processing, with data averaged over time (350–750 ms) and frequencies, separately for low frequency range (5–28 Hz) and gamma-band range (63–80 Hz). Blue connections express a decrease and red connections an increase in strength of connectivity between groups. Thalamo-cortical connectivity was significantly decreased in the lower frequency range for ketamine (*d* = 1.21) and Schizophrenia group (*d* = 0.98), and was significantly stronger in the high frequency range for all groups (ketamine *d* = 0.71, FEP *d* = 1.26, schizophrenia *d* = 0.93). Cortico-cortical connectivity, however, was decreased following ketamine infusion in both the low- (*d* = 1.42) and high-frequency range (*d* = 0.64), but was increased in the low-frequency range in patients with schizophrenia (*d* = 1.24), and increased in the higher range in both FEP (*d* = 1.40) and schizophrenia (*d* = 1.01) patients. CUN = cuneus; CAL = calcarine fissure; LING = lingual gyrus; PCG = posterior cingulate gyrus; PCUN = precuneus; SOG/MOG/IOG = superior/middle/inferior occipital gyrus; THA = thalamus.

Second-level ANOVAs, using z-normalized data, revealed a main effect of Group for both lower frequencies [5–28 Hz: *F*(2,37) = 3.7, *P = *0.035] and higher frequencies [63–80 Hz: *F*(2,37) = 24.2, *P < *0.0001]. Results from *post hoc* comparisons indicated that in the lower frequency range, this was due to significant power differences between ketamine and FEP patients (*P = *0.016) and between both patient groups (FEP versus schizophrenia: *P = *0.026), respectively. In the 63–80 Hz range, ketamine effects on power estimates differed significantly from those seen in both FEP (*P < *0.0001) and schizophrenia patients (*P < *0.0001).

#### Functional connectivity in chronic schizophrenia and FEP

Granger causality patterns also differed from ketamine effects by showing predominantly increased connectivity in both FEP and chronic schizophrenia patients compared to controls (Fig. 4, Table 3 and Supplementary Table 2). In the FEP group, alterations in Granger causality connectivity involved only higher frequency range thalamo-cortical and cortico-cortical interactions. Increased thalamo-cortical signalling (*d* = 1.26) was observed between the thalamus and middle occipital gyrus. Moreover, increased cortico-cortical connectivity (*d* = 1.40) was present between precuneus and superior occipital, inferior occipital and lingual gyrus, and between the precuneus and middle occipital gyrus.

Similar to FEP patients, data from patients with chronic schizophrenia were characterized by increased Granger causality connectivity in both cortico-cortical and thalamo-cortical circuits. In contrast to FEP patients, however, it included also significant upregulations in the lower-frequency range. Thalamo-cortical hyper-connectivity in both frequency ranges included connections primarily with the precuneus (*d* = 0.98). Significantly upregulated thalamo-cortical connectivity in the high-frequency range (*d* = 0.93) included connections with the posterior cingulate gyrus and lingual gyrus. Significant cortico-cortical hyper-connectivity included mainly connections to and from the precuneus and posterior cingulate gyrus with both striate (calcarine fissure, cuneus, lingual gyrus) and extrastriate cortical regions (inferior, middle and superior occipital gyrus), with upregulations present in both frequency ranges (low: *d* = 1.24, high: *d* = 1.01). Further examination revealed that 24 of 37 connections that were altered in chronic schizophrenia involved the posterior cingulate gyrus (65% of connections).

Analyses of feedforward and feedback directionality of connectivity revealed that in the schizophrenia group, both feedforward and feedback connections were equally upregulated at low and high frequencies. For the FEP group, thalamo-cortical interactions involved preferentially feedforward processes while for cortico-cortical connectivity both feedforward and feedback communication was changed.

#### Baseline differences

We statistically tested baseline power (−500 to −100 ms) in all 18 AAL nodes and all frequencies between 1 and 90 Hz, using Monte Carlo-based permutations and *t*-test statistics (cluster-corrected for multiple comparisons) to assess whether differences in post-stimulus activity between ketamine versus placebo conditions as well as for comparisons involving chronic schizophrenia patients and FEP groups versus controls were influenced by alterations in baseline activity. For all comparisons, there were no significant differences observed.

### Correlations: behavioural and psychopathology versus MEG spectral power and connectivity data

Pearson’s correlations were used on z-normalized data to investigate relationships between changes in spectral power and Granger causality connectivity changes in the low (5–28 Hz) and high frequency (63–80 Hz) range with response times, detection rates and PANSS ratings (Supplementary Table 3 and Supplementary Fig. 1). Bootstrapping (1000 randomizations) was applied to control for spurious findings.

In the ketamine condition, reduced response accuracy correlated with increased occipital gamma-band power (*r* = − 0.55, *P = *0.041), but such a pattern was not observed in the patient groups. Furthermore, a dissociation between ketamine and the FEP/schizophrenia groups was found for response times, which correlated negatively with the gamma-band effect in the FEP group (*r* = − 0.77, *P = *0.009) whereas the ketamine condition showed a positive correlation.

Increased thalamo-cortical and cortico-cortical high-gamma Granger causality connectivity in the FEP group correlated positively with response times (thalamo-cortical: *r* = 0.71, *P = *0.002, cortico-cortical: *r* = 0.60, *P = *0.048), whereas ketamine did not affect behaviour–connectivity relationships. In the lower frequency range, upregulated lower frequency power as well as increased thalamo-cortical connectivity in the schizophrenia group correlated with decreased accuracy (power: *r* = − 0.56, *P = *0.023, connectivity: *r* = − 0.53, *P = *0.034).

We also systematically explored the relationship between PANSS scores, spectral power and connectivity changes. The PANSS hallucination item ‘P3’ showed a significant negative correlation with gamma power in the FEP group (*r* = − 0.68, *P = *0.003) and with high-gamma thalamo-cortical connectivity upregulation following ketamine administration (*r* = − 0.69, *P = *0.006). On the other hand, PANSS Cognitive and Positive score increases following ketamine challenge correlated negatively with low frequency (5–28 Hz) cortico-cortical connectivity decreases (Cognitive: *r* = − 0.57, *P = *0.033, Positive: *r* = − 0.48, *P = *0.050).

## Discussion

The current study attempted to systematically explore the commonalities between the effects of a sub-anaesthetic dose of ketamine on neural oscillations during visual processing in thalamo-cortical networks and alterations in chronic schizophrenia and FEP patients. Recent evidence has highlighted the crucial contribution of thalamo-cortical interactions towards the pathophysiology of schizophrenia (Woodward *et al.*, 2012; Anticevic *et al.*, 2015*b*), which is supported by extensive evidence on the impact of NMDAR hypofunctioning on thalamo-cortical circuits (Santana *et al.*, 2011; Dawson *et al.*, 2013; Hoflich *et al.*, 2015; Anderson *et al.*, 2017; Pratt *et al.*, 2017) and the relationship between NMDARs and feedback processing during visual perception (Self *et al.*, 2012; van Loon *et al.*, 2016).

Our findings could not support the main hypotheses. While our data demonstrate that ketamine profoundly impacts upon the power and spectral interactions of neural oscillations in thalamo-cortical networks, the effects of ketamine on both the amplitude of gamma-band oscillations as well as on functional connectivity were largely opposite to the alterations observed in both FEP and chronic schizophrenia patients, suggesting that the acute NMDA hypofunction may not fully account for altered neurodynamics in the disorder.

Consistent with previous reports (Krystal *et al.*, 1994), ketamine administration led to an increase in psychopathology as well as reduced detection rates and elevated response times in healthy volunteers. Increased PANSS ratings and impaired behavioural performance were accompanied by an increase in 60–80 Hz power in occipital cortices. These findings are consistent with data from auditory perception (Hong *et al.*, 2010), visual cortex (Shaw *et al.*, 2015) and resting state MEG recordings (Rivolta *et al.*, 2015), indicating that ketamine upregulates rhythmic activity at gamma-band frequencies.

Moreover, we found elevated theta, alpha and beta power, which is typically suppressed during visual tasks, suggesting a disinhibition of neural circuits during ketamine. This effect was prominent not only in occipital regions but also in thalamus, a region with a high concentration of NMDARs (Monaghan and Cotman, 1985). In addition to this finding, ketamine also increased 1–5 Hz oscillations in the right thalamus, which has been observed in invasive electrophysiological studies (Zhang *et al.*, 2012). While observations in deep brain structures with MEG can be challenging because of the decay of the magnetic field, the large effect size of this modulation in our data (Table 4) and the overlapping spectral profile of thalamic channels with cortical generators support the feasibility of MEG in combination with individual anatomical information to assess thalamic signals (Attal *et al.*, 2007; Roux *et al.*, 2013).

One novel observation of the current study is the examination of changes in directed, spectral interactions following ketamine administration. Recent evidence suggests that frequency-specific, hierarchical networks can be reconstructed from MEG data (Michalareas *et al.*, 2016), which mirror findings obtained from invasive electrophysiological recordings (Bastos *et al.*, 2015). Accordingly, this approach allowed us to determine whether ketamine impacted specifically on feedback communication given the contribution of NMDARs in this process (Self *et al.*, 2012) and the role of beta/alpha-band oscillations in feedback processing during visual and attentional tasks (Buschman and Miller, 2007; van Kerkoerle *et al.*, 2014; Michalareas *et al.*, 2016).

Our data, however, only partially support this framework. Overall, we found that ketamine reduced both feedforward and feedback communication especially at lower frequencies, while in the 63–80 Hz frequency range, enhanced thalamo-cortical connectivity with parietal and early visual cortex was found. In contrast, cortico-cortical was decreased across a wide range of occipital-parietal connections.

Importantly, the analysis of MEG data from a sample of FEP and chronic schizophrenia patients revealed a strikingly different pattern both in regards to the direction of effects in spectral power and connectivity (Table 4 and Supplementary Table 2). Consistent with prior data highlighting an impairment in the generation of gamma-band oscillations during auditory (Kwon *et al.*, 1999) and visual paradigms (Spencer *et al.*, 2003; Sun *et al.*, 2013), both FEP and schizophrenia groups showed a pronounced reduction in the 50–80 Hz power range in occipital cortex. Further studies need to address the question, however, whether there is a causal link between impaired gamma-band oscillations and cognitive deficits in schizophrenia, for example, through the use of frequency-specific brain stimulation (Thut *et al.*, 2011).

For low frequency power (5–28 Hz), we found that both the ketamine condition as well as the chronic schizophrenia group showed a comparable upregulation of spectral power. During sensory processing, activity at theta, alpha and beta frequencies are typically suppressed, indicating desynchronization to facilitate processing of relevant stimulus information (Pfurtscheller and Lopes da Silva, 1999; Hoogenboom *et al.*, 2006). The finding in chronic schizophrenia patients suggests that this mechanism is impaired, which could potentially interfere with processing of relevant stimulus material as indicated by the negative relationship between accuracy and low frequency power (Supplementary Table 3).

Overall, the differential findings in regards to gamma-band power and low frequency activity suggest that impairments in high frequency activity could represent a trait marker in schizophrenia as both FEP and chronic schizophrenia patients were characterized by large reductions in spectral power. In contrast, it is conceivable that low frequency oscillations are a state marker that is influenced by clinical symptoms, which expectedly differed between FEP and chronic schizophrenia groups.

There were also notable differences between clinical groups with regards to the pattern of directed connectivity. In the schizophrenia group, Granger causality patterns were exclusively characterized by hyperconnectivity that involved both feedforward and feedback connections at low and high frequencies, highlighting a profound dysregulation of neuronal communication. These alterations seemed to involve preferentially connections with the posterior cingulate gyrus, a prominent node of the default mode network (DMN) (Raichle *et al.*, 2001), which is typically deactivated during task-relevant processing. Previous evidence from functional MRI data in schizophrenia patients suggests that the switching between the DMN and attention networks is impaired (Anticevic *et al.*, 2012*a*).

Compared to patients with chronic schizophrenia, alterations in Granger causality connectivity involved only the gamma-band range, which showed a mixture of thalamo-cortical and cortico-cortical interactions. Together with the absence of alterations in low-frequency power in FEP-patients, these findings suggest that there may be illness-specific alterations in neuronal dynamics that could potentially indicate a progressive dysfunction during the course of illness. However, we would like to note that these findings need to be replicated in larger samples.

Importantly, neither the alterations in task-related oscillations following ketamine administration nor the power changes in the patient samples were driven by differences in baseline activity. This contrasts with previous findings in preclinical studies of NMDAR antagonists on neural oscillations (Ehrlichman *et al.*, 2009; Saunders *et al.*, 2012) as well as with EEG studies in schizophrenia (Hirano *et al.*, 2015) that have implicated alterations in baseline gamma-band activity for sensory processing deficits in schizophrenia.

Taken together, these data highlight the importance of NMDAR mediated neurotransmission for the amplitude and organization of spectral organization of task-related neural oscillations. Excitation and inhibition balance is considered a crucial factor for the generation of neural oscillations and interactions in hierarchical networks (Yizhar *et al.*, 2011). Consistent with a wealth of preclinical data (Pinault, 2008; Sivarao *et al.*, 2016), the current findings highlight that the NMDAR hypofunctioning enhances the amplitude of gamma-frequency activity and upregulates low frequency oscillations while behavioural performance is reduced. Accordingly, optimal information processing in large-scale networks can be disrupted both by an excess of gamma-band activity as well as by an impairment in the generation of gamma-band oscillations as observed in schizophrenia.

The current data have implications not only for understanding the mechanisms of spectral interactions in large-scale networks during normal brain functioning but also for the role of NMDAR hypofunctioning in the pathophysiology of schizophrenia. We would like to note that the impact of ketamine on the amplitude as well as spectral interactions during task-related gamma-band oscillations is not compatible with visually-elicited neural oscillations in patients at an early stage of psychosis as well as in a sample of chronic schizophrenia patients. Previous preclinical (Schobel *et al.*, 2013), resting state functional MRI data (Anticevic *et al.*, 2015*a*) and EEG studies (Umbricht *et al.*, 2000; Schmidt *et al.*, 2013; Kort *et al.*, 2017) have suggested that the neural signatures induced by acute NMDA antagonists, such as ketamine, resemble behavioural deficits and physiological alterations observed in schizophrenia, in particular during early stages of the disorder. The current data do not support this hypothesis and suggest instead that alterations in task-related oscillations in schizophrenia, in particular at gamma-band frequencies, may be not compatible with the effects of acute NMDAR hypofunctioning.

There are several limitations of this study that relate to the sample size of healthy volunteers and patients with schizophrenia or FEP as well as our pharmacological manipulation. First, we would like to note that while the study used only moderate samples of participants, the effect sizes for ketamine effects on power and spectral interactions as well for differences between controls and patient populations were consistently large (Table 4 and Supplementary Table 3). Moreover, statistical contrasts for main effects survived conservative statistical correction. Finally, we would like to emphasize the consistent effects of the visual paradigm used across groups on the modulation of gamma-band oscillations with high signal-to-noise and excellent test-rest reliability (Tan *et al.*, 2016).

Second, it is important to note that ketamine while acting on NMDARs also leads to a dysregulation of serotonergic (Li *et al.*, 2015) and dopaminergic neurotransmission (du Jardin *et al.*, 2016). Accordingly, it is conceivable that the changes in both spectral power and functional connectivity observed in the current study are not only due to NMDA hypofunctioning. We believe, however, that there is evidence to suggest that the effects of ketamine on gamma-band oscillations, for example, are indeed strongly mediated by NMDARs as the effects of ketamine on gamma-band oscillations are the result of specific actions on NMDAR subunits (Kocsis, 2012) and serotonergic antagonists, for example, do not lead to a similar dysregulation of high frequency activity following NMDAR hypofunctioning (Wood *et al.*, 2012).

Finally, we would like highlight that the mechanisms underlying the upregulation of gamma-band oscillations following the administration of ketamine remain to be determined. As highlighted previously, several scenarios have been proposed that include the downregulation of NMDARs on parvalbumin-positive interneurons that could elevate the contribution of faster AMPA receptors onto parvalbumin-positive cells, providing in turn favourable conditions for the generation of gamma-band oscillations (Rotaru *et al.*, 2011). Further, upregulation of gamma-band oscillations following ketamine administration could also target other subclasses of interneurons (Jadi *et al.*, 2016) as well as presynaptic effects of NMDARs (Pafundo *et al.*, 2018). However, differentiation of these mechanisms will require further careful investigations into the interaction between NMDARs, different interneuron populations as well as the contribution of other neurotransmitter systems that could not only be relevant for the understanding the role of NMDAR hypofunctioning in schizophrenia but also for the generation and mechanistic importance of neural oscillations in general.

In summary, our findings strongly support the ability of acute ketamine to induce schizophrenia-like symptoms and neurocognitive deficits. In addition, many measures such as low frequency oscillatory changes in both occipital cortex and thalamus show changes in the same direction following acute ketamine treatment versus placebo and schizophrenia versus control. However, our findings also highlight the dissociation between changes in gamma-band oscillations observed following acute ketamine administration and those seen in schizophrenia. These findings suggest that the reductions in gamma-band activity observed in both patients with FEP and those with chronic schizophrenia cannot be attributed to acute NMDA receptor blockade, but instead may reflect consequences of longer term NMDAR dysfunction (McNally *et al.*, 2013; Ahnaou *et al.*, 2017), or effects of ketamine mediated via non-NMDA neurotransmitter systems.

## Funding

The project was supported by the LOEWE grant Neuronale Koordination Forschungsschwerpunkt Frankfurt (NeFF). This study was also supported by the project MR/L011689/1 from the Medical Research Council (MRC).

## Competing interests

P.J.U. has received research support from Lilly and Lundbeck. S.M.L. has received lecture fees from Janssen, Otsuka and Sunovion. The remaining authors report no biomedical financial interests or potential conflicts of interest.

## Supplementary material

Supplementary material is available at *Brain* online.

## Supplementary Material

Supplementary Data

## Abbreviations

FEP: first-episode of psychosis
MEG: magnetoencephalography
NMDA(R): *N*-methyl-d-aspartate (receptor)
PANSS: Positive and Negative Syndrome Scale
