# Supplementary material for: Acute ketamine dysregulates task-related gamma-band oscillations in thalamo-cortical circuits in schizophrenia
Source: Brain. 2018 Jul 17;141(8):2511–26. doi: 10.1093/brain/awy175 (PMC6061682; doi:10.1093/brain/awy175)

SI-Table 1. Node-node pairs with significant effects in Granger Causality (GC) estimates for LOWFREQ (5-28 Hz) and HIGHFREQ (63-80 Hz) activity, separately for CONDITION effect (KETAMINE vs. PLACEBO) or GROUP effects (ScZ/FEP vs. CONTROLS)

	Sign GC connections	*t*-values	*p*-values
**KETAMINE vs. PLACEBO (n=14)**			
LOWFREQ (5-28 Hz)			
Thalamo-cortical Feedforward DAI:0.54-0.83	RPCUN-RTHA, LPCG-RTHA, LPCUN-LTHA	-2.2 to -2.9	0.0211 to 0.0132
Thalamo-cortical Feedback DAI: -0.49	RTHA-RIOG	-3.4	0.0001
Cortico-cortical Feedforward DAI: 0.17-0.68	LLING-RIOG, LCUN-LCAL, LPCG-RIOG, LSOG-LLING, LSOG-LCAL, LIOG-RIOG	-2.0 to -3.0	0.0226 to 0.0074
Cortico-cortical Feedback DAI:	LMOG-RCUN, LMOG-RPCUN, LSOG-LCUN, LCAL-LCUN, LMOG-RLING, RIOG-LPCUN, LPCUN-RSOG, LMOG-RCAL, LIOG-LCAL, LMOG-RSOG, RLING-LCUN, RIOG-LSOG, LMOG-RPCG, RSOG-RCUN	-2.0 to -3.3	0.0271 to 0.0001
HIGHFREQ (63-80 Hz)			
Thalamo-cortical Feedforward DAI: 0.95	RCAL-LTHA	2.5	0.0198
Thalamo-cortical Feedback DAI: -0.68	RTHA-RPCUN	2.8	0.0130
Cortico-cortical Feedforward DAI: 0.42 to 0.86	LSOG-LPCUN, LLING-LPCUN, LIOG-LPCUN	-2.0 to -2.2	0.0213 to 0.0031
Cortico-cortical Feedback DAI: -0.16	RIOG-LMOG	-2.2	0.0054
**FIRST-EPISODE vs. CONTROLS (n=10)**			
HIGHFREQ (63-80 Hz)			
Thalamo-cortical Feedback DAI: 0.18	LMOG-RTHA	2.8	0.0062
Cortico-cortical Feedforward DAI: 0.06 to 0.74	RSOG-LPCG, RSOG-LIOG	2.5 to 3.2	0.0077 to 0.0043
Cortico-cortical Feedback DAI: -0.64	RMOG-RPCUN	3.0	0.0074
**SCHIZOPHRENIA vs. CONTROLS (n=16)**			
LOWFREQ (5-28 Hz)			
Thalamo-cortical Feedforward DAI: 0.60	LPCUN-LTHA, RPCUN-LTHA	2.3 to 2.9	0.0190 to 0.0030
Cortico-cortical Feedforward DAI: 0.01-0.17	RPCG-RIOG, RPCG-LPCUN, RPCG-LMOG, LPCG-RPCUN	2.2 to 2.5	0.0170 to 0.0100
Cortico-cortical Feedback DAI: -0.10 to -0.80	LPCUN-RCUN, RPCUN-LMOG, RPCG-RCAL, RPCG-RMOG, LPCG-RSOG, LPCUN-RMOG, LPCG-LCAL, LPCUN-RLING, LPCUN-RCAL, LPCG-RIOG, LPCG-RLING, RPCG-RLING, LLING-LCAL, LPCG-RCAL, LPCG-RMOG	2.3 to 3.7	0.0210 to 0.0010
HIGHFREQ (63-80 Hz)			
Thalamo-cortical Feedforward DAI: 0.50 to 0.81	RPCG-RTHA, LPCUN-LTHA, RPCUN-LTHA, RPCG-LTHA	2.2 to 2.4	0.0200 to 0.0070
Thalamo-cortical Feedback DAI: -0.93	RTHA-RLING	2.1	0.0180
Cortico-cortical Feedforward DAI: 0.22 to 0.42	RPCG-LMOG, LPCG-RSOG	2.3 to 2.4	0.0160 to 0.0080
Cortico-cortical Feedback DAI: -0.22 to -0.78	RPCUN-RSOG, RPCG-LSOG, RPCG-LCUN, RPCUN-LMOG, RPCG-LLING, LPCG-RSOG, LPCG-RCUN, LPCG-RLING, RPCG-RLING, LPCG-RIOG	2.1 to 2.8	0.0250 to 0.0040

Abbreviations: CAL = calcarine sulcus, CUN = cuneus, LING = lingual gyrus, SOG = superior occipital gyrus, MOG = middle occipital gyrus, IOG = inferior occipital gyrus, PCG = posterior cingulate cortex, PCUN = precuneus, THA = thalamus. L = left hemisphere, R = right hemisphere. DAI = Directed Asymmetry Index (negative values represent predominantly feedback, whereas positive values indicate predominantly feedforward connectivity within the node-node pair).

SI-Table 2. Cohen's *d* effect sizes for group and condition comparisons

	KETAMINE vs PLACEBO	FIRST-EPISODE vs CONTROLS	SCHIZOPHRENIA vs CONTROLS
**Oscillatory Power changes**			
Occipital cortex: 63-80 / 53-76 / 62-83 Hz	⇧ 0.88	⇩ 1.49	⇩ 1.07
Occipital cortex: 5-28 / 7-23 Hz	⇧ 1.12	–	⇧ 1.06
Left Thalamus: 6-20 / 7-20 Hz	⇧ 1.31	–	⇧ 1.07
Right Thalamus: 6-30 / 8-20 Hz	⇧ 0.99	–	⇧ 1.02
**GC connectivity changes**			
LOWFREQ (5-28 Hz)			
Thalamo-cortical feedforward	⇩ 0.81	–	⇧ 0.98
Thalamo-cortical feedback	⇩ 0.90	–	–
Cortico-cortical feedforward	⇩ 1.33	–	⇧ 1.10
Cortico-cortical feedback	⇩ 1.06	–	⇧ 1.22
HIGHFREQ (63-80 Hz)			
Thalamo-cortical feedforward	⇧ 0.77	–	⇧ 0.91
Thalamo-cortical feedback	⇧ 0.68	⇧ 1.26	⇧ 0.74
Cortico-cortical feedforward	⇩ 0.81	⇧ 1.38	⇧ 1.12
Cortico-cortical feedback	⇩ 0.59	⇧ 1.32	⇧ 0.91

SI-Table 3. Pearson's Correlations between behavioral performance, psychopathology and neuronal changes in low- and high-frequency power and connectivity

	**Behavior**		**PANSS subscale item scores**						
	**mRT**	**ACC**	**NEG**	**EXC**	**COG**	**POS**	**P3**	**DEP**	**TOT**
**KETAMINE vs PLACEBO**									
(5-28 Hz) power ⇧	0.25	0.10	0.08	0.16	-0.11	-0.03	0.17	0.21	-0.05
(63-80 Hz) power ⇧	0.41	**-0.55***	0.12	0.05	0.52	0.21	-0.05	0.02	0.23
(5-28 Hz) GC-TC ⇩	0.23	0.42	-0.04	0.07	0.32	0.46	-0.28	0.13	-0.10
(5-28 Hz) GC-CC ⇩	0.12	0.28	-0.16	-0.05	**-0.57***	**-0.48***	-0.44	-0.08	-0.42
(63-80 Hz) GC-TC ⇧	-0.38	-0.23	-0.41	-0.38	0.01	-0.16	**-0.69***	-0.28	0.36
(63-80 Hz) GC-CC ⇩	0.41	-0.28	0.24	-0.23	-0.36	-0.44	-0.28	-0.27	-0.15
**FIRST-EPISODE vs CONTROLS**									
(5-28 Hz) power (⇧)	0.06	0.24	0.19	0.26	0.36	0.43	-0.13	0.03	0.39
(63-80 Hz) power ⇩	**-0.77***	0.48	0.09	0.29	0.39	0.12	**-0.68***	-0.38	-0.36
(63-80 Hz) GC-TC ⇧	**0.71***	-0.56	-0.14	-0.09	-0.12	-0.40	-0.32	-0.25	-0.31
(63-80 Hz) GC-CC ⇧	**0.60***	-0.25	0.07	0.11	-0.06	0.23	0.07	-0.02	0.09
**SCHIZOPHRENIA vs CONTROLS**									
(5-28 Hz) power ⇧	-0.21	**-0.56***	0.05	**0.55***	0.46	0.33	0.18	0.16	0.35
(63-80 Hz) power ⇩	-0.22	0.46	-0.12	-0.01	-0.25	0.11	0.04	0.12	-0.02
(5-28 Hz) GC-TC ⇧	-0.17	**-0.53***	-0.19	-0.03	0.21	-0.05	0.01	0.20	0.11
(5-28 Hz) GC-CC ⇧	-0.30	-0.43	-0.08	-0.01	0.19	-0.13	-0.08	0.02	-0.03
(63-80 Hz) GC-TC ⇧	-0.45	-0.30	-0.23	-0.01	0.20	0.05	0.01	0.04	-0.06
(63-80 Hz) GC-CC ⇧	-0.40	-0.10	-0.04	0.19	0.25	-0.02	-0.06	0.23	0.11

** Pearson's r values with significant p-values below 0.05(bootstrapped corrected n=1000)*

Abbreviations: GC-TC = Granger Causality mean values across significant Thalamo-Cortical node-node pairs, GC-CC = Granger Causality mean values across significant Cortical-Cortical node-node pairs, mRT = mean response times, ACC = accuracy (% correct responses), PANSS subscales: NEG = Negative, EXC = Excitement, COG = Cognitive, POS = Positive, P3 = Hallucinatory behavior (subitem of POS scale), DEP = Depression, TOT = Total scores.

SI-Figure 1. Significant Pearson's Correlations between behavioral performance, psychopathology and neuronal changes in low- and high-frequency power and connectivity

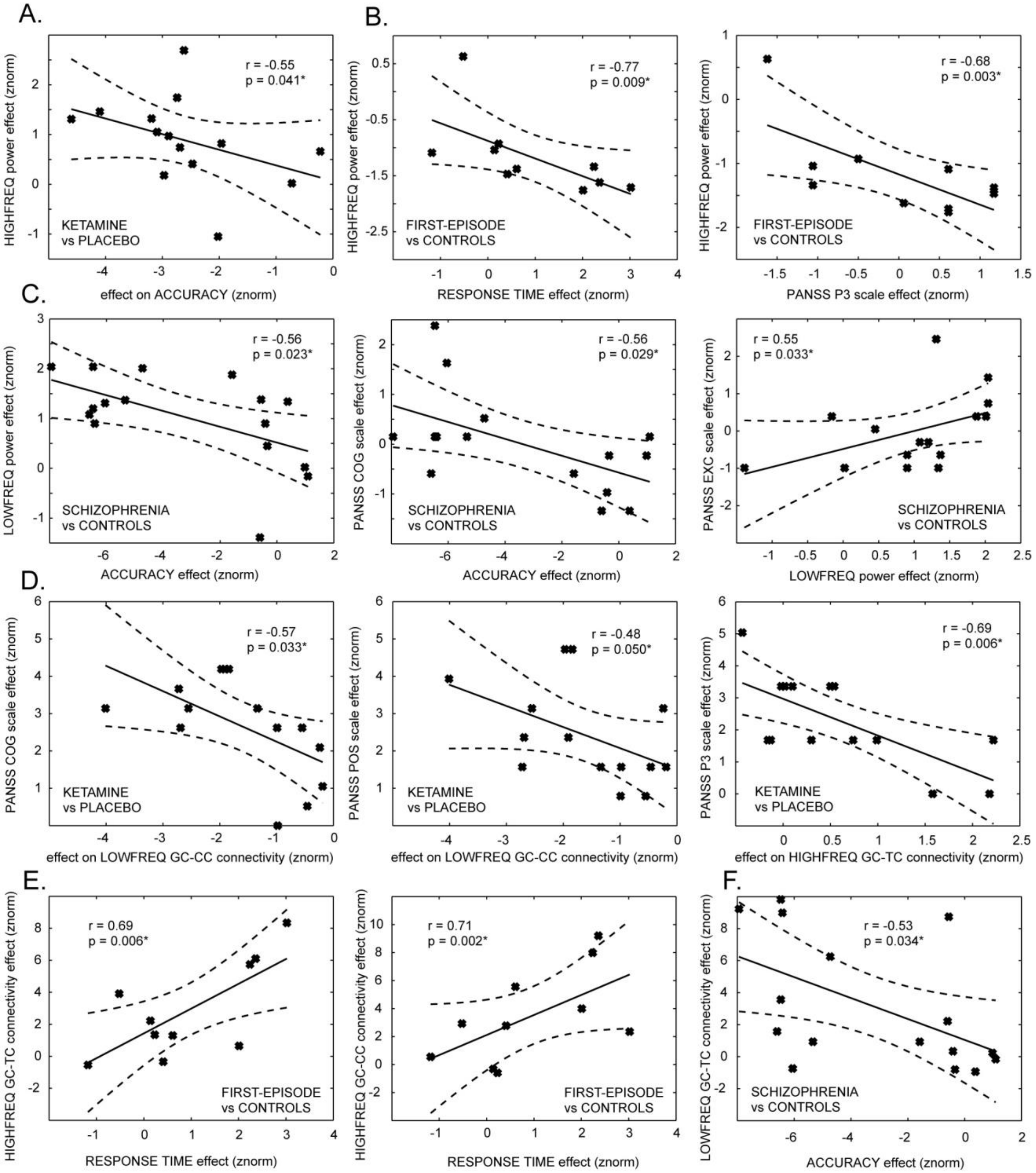

Supplement: Supplementary Data [file awy175_brain-2017-02269-file010.pdf]
